# Wine By-Products as Raw Materials for the Production of Biopolymers and of Natural Reinforcing Fillers: A Critical Review

**DOI:** 10.3390/polym13030381

**Published:** 2021-01-26

**Authors:** Alessandro Nanni, Mariafederica Parisi, Martino Colonna

**Affiliations:** Department of Civil, Chemical, Environmental and Material Engineering, University of Bologna, Via U. Terracini 28, 40131 Bologna, Italy; mariafederica.paris2@unibo.it (M.P.); martino.colonna@unibo.it (M.C.)

**Keywords:** wine by-products, biopolymers, bio-composites, bio-fillers

## Abstract

The plastic industry is today facing a green revolution; however, biopolymers, produced in low amounts, expensive, and food competitive do not represent an efficient solution. The use of wine waste as second-generation feedstock for the synthesis of polymer building blocks or as reinforcing fillers could represent a solution to reduce biopolymer costs and to boost the biopolymer presence in the market. The present critical review reports the state of the art of the scientific studies concerning the use of wine by-products as substrate for the synthesis of polymer building blocks and as reinforcing fillers for polymers. The review has been mainly focused on the most used bio-based and biodegradable polymers present in the market (i.e., poly(lactic acid), poly(butylene succinate), and poly(hydroxyalkanoates)). The results present in the literature have been reviewed and elaborated in order to suggest new possibilities of development based on the chemical and physical characteristics of wine by-products.

## 1. Introduction

The plastic industry is today facing a green and economic revolution to maintain its indispensable and predominant role within everyday human life. Conventional plastics have started to be discouraged because they derive from petrochemical sources and because they are largely responsible for maritime pollution. In 2018, global CO_2_ emissions amounted to almost 34 billion tons [1], and oil and gas companies, consuming about the 57% of global fuels for their plants, emitted more than 50% of the total greenhouse gasses (GHGs) [2,3]. In addition, at the present consumption rate, crude oil and natural gas are believed to have a life expectancy of just 50 years [1]. Thus, fossil fuels price are increasing, and since 5–7% of these sources are destined for synthesis of polymer building blocks, conventional plastics are also starting to become more expensive [4] and less attractive to investors. From another side, plastic pollution is increasingly becoming one of the main environmental problems of our era. Despite the efforts made to promote mechanical recycling, energy recovery, and the reuse of plastics, in 2015 almost 55% of plastic wastes were destined to inadequate landfills, which are often open and/or uncontrolled, and lead to plastic wastes entering the oceans [5]. It is estimated that around 270 thousand tons of plastic wastes are today floating in the oceans’ surfaces [5,6] and that micro and nano-plastics, assimilated within the gut or the tissue of small marine organisms [7], are now reaching the human food chain level [8], as confirmed by the increase of documented cases of plastic ingestion and plastic inhalation [9,10].

It is within this complex economic and environmental context that in the last decades, because of their ability to theoretically replace fossil fuels and/or mitigate plastic pollution, bioplastics—defined as bio-based and/or biodegradable polymers—have started to gain a central role in both scientific academia and industry. Nevertheless, as reported in Table 1, in 2018 the production of bioplastics was only 2.1 Mt (1.2 Mt of bio-based polymers and 0.9 Mt of biodegradable polymers), which barely represents 0.6% of the total produced polymers [11,12]. This limited production is mainly due to the low yields of conversion (from biomass to bio-based building block) and to the technological barriers present for these chemical or biological transformations. As a consequence, the final price of biopolymers is generally higher compared to those of conventional polymers [13,14,15]. In addition, bio-based building blocks are mainly obtained from first generation feedstocks such as wheat, corn, rapeseed, or sugar cane which, being food-competitive, are expensive and ethically questionable. Therefore, in recent years, the use of non-edible second-generation feedstocks, such lignocellulosic crops and agricultural residues and by-products, is increasingly gaining importance, since they would decrease the final prices of biopolymers [16] and would set down moral concerns. Moreover, the valorization of these wastes would also represent an alternative solution to the disposal management problems of agro-industrial farms. Following an alternative approach, research is also focusing on the use of natural wastes as reinforcing and cost-advantage bio-fillers and/or bio-fibers. In this way, the final price of biopolymers would be decreased almost proportionally to the natural filler content that generally ranges from 10 up to 50 %wt., and the resultant bio-composite would have new attractive and enhanced properties. In addition, the use of natural fillers would maintain or even enhance the bio-based content of the biopolymers to which they are mixed. Finally, the possibility to utilize agro-wastes as different polymer additives is today investigated. In fact, despite that these products are usually mixed within plastics in low amounts, polymer additives, such as stabilizers (1.3 Mt [17]) and plasticizers (6.4 Mt [18]), are globally produced in high amounts and their green alternatives would therefore benefit the environment. 

Therefore, the aim of this review is to show the potential uses of wine by-products as feedstocks for the production of bio-based building blocks or biopolymers directly synthetized by microorganisms (e.g., poly(hydroxyalkanoates) (PHAs)) and for the fabrication of cost-effective reinforcing fillers. In each case, this review particularly focuses on the principal biopolymers both bio-based (fully or partially) and biodegradable, and present in large-scale, which are namely poly(lactic acid) (PLA), poly(butylene succinate) (PBS), and poly(hydroxyl butyrate) (PHB). The results presented in the literature have been reported and discussed, and a possible route of valorization of wine by-products within the polymer sector has been proposed. Despite the recent publication of the *Handbook of Grape Processing By-Products*, edited by C.Galanakis in 2017 [19], and despite the presence in literature of other reviews dealing with the new trends of valorization of wine waste [20,21,22,23,24], there are no reviews fully focused on the potentialities of wine by-products as raw materials for the plastic industry. 

## 2. Description of the Winemaking By-Products

### 2.1. Wine By-Products in Numbers

**Figure 1:**
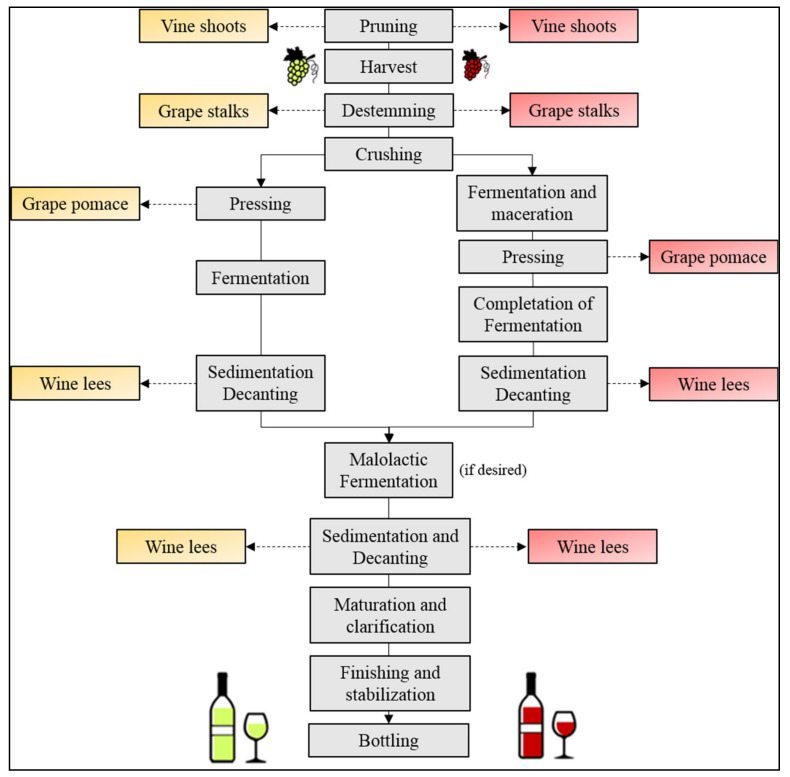
Scheme of the winemaking processes and associated solid wine wastes.

Wine is one of the most produced beverages in the world and in 2018 its global production was around 29.2 million hectoliters (Mhl) [25]. In 2018, with 57% of the total production, Europe was the leading producer, followed by the Americas (26%), Asia (7.5%), Oceania (5.4%), and Africa (4.5%). Meanwhile, in terms of countries, the top five wine producers have been Italy (18.8% of the total production), France (16.6%), Spain (15.2%), USA (8.2%), and Argentina (5.0%). Considering grape, the fourth most cultivated fruit in the world after banana, watermelon, and apple, its production in 2018 was 77.8 Mt [26], and the leading producer countries were China (11.7 Mt), Italy (8.6 Mt), USA (6.9 Mt), Spain (6.9 Mt), and France (6.2 Mt). Globally, 57% of cultivated grapes have been used for the winemaking processes (including musts and juices), 36% have been destined to tables (table grape), and 7% have been converted to dried grapes [26]. From these data, it can be calculated that to produce 1 L of wine, an average 1.5 kg of grapes is needed, or analogously, that wine yield is of 66% (it generally ranges between 65% to 75%, depending on the wine typology). Thus, assuming a mean wine density of 1 kg/L [27], it can be calculated that winemaking processes generate approximately 0.3–0.5 kg of wine by-products for each liter of wine produced, to which must be added the by-products generated during the pruning season (winter). In fact, following the scheme reported in Figure 1, vine shoots (or vine trimmings) and wine leaves are the first wine by-products, which are obtained from the in-field interventions aimed to preserve the grapevine reproduction. Subsequently, in harvest season (August–November), mature grapes are collected and sent to the winemaking companies in which the destemming step, carried out for both red and white wines (and juices), generates other by-products called stalks (or stems). At this point, grapes are pressed to extract the grape juice and the residue grape pomace (or grape marc), formed by grape seeds and grape peels in 1:1 (*w/w*) ratio, is obtained. In the case of white wines (or juices), grape pomace is immediately discarded from the pressed juice (now called must), meanwhile, in the case of red wine, grape pomace is initially left with the must for a certain time of the fermentation period to extract pigments and grape constituents and successively removed and discarded. Finally, after the completion of alcoholic (and in some cases malolactic) fermentations, residues called wine lees are generally collected during the operations of decanting, clarification, tartaric stabilization, and/or filtration. Summarizing, the main solid winemaking by-products are vine shoots, grape stalks, grape pomace (formed by grape peels and grape seeds, approximatively 1:1 weight ratio), and wine lees. Considering that the global area under vines in 2018 was 7.4 million hectares (Mha) [26] and that vine shoots are generated with a rate of 1.4–2 ton/ha [28], independently from the usage of the grapes (grape table, wines, dried), it can be stated that they are the most produced wine by-products. Instead, grape pomaces, being 20–25% of the processed grapes, represent the most abundant fraction of by-products generated during winemaking processes followed by wine lees (2–6%) and grape stalks (3–5%) (Table 2). 

Until recently, these wine wastes have been generally destined to distillation, landfilling, incineration, and/or land-spread. In particular, grape stalks have been mainly land-spread (76% in Italy, 55% in Spain, and 40% in France), disposed in landfill (50% in Greece), or destroyed by incineration (36% in France). Grape pomaces have been mainly used as feed for distillation (100% Italy, 90% France, 30–60% Spain) or for land spreading (50% in Spain), while in Greece they are mainly discarded to landfill (67%) or sold as animal fodder (33%). Finally, vine shoots have been commonly land-spread or burned in the field and wine lees have been generally distilled in all considered countries.

Nevertheless, these conventional uses of the wine wastes have been severely reconsidered in the past years. As an example, because of their low pH, high organic matter, and high concentrations of macronutrients, wine wastes are not ideal fertilizers without expensive pre-treatments or conditioning steps [29]. Similarly, wine by-products can inhibit or modify the germination properties when used as amendments and landfilling is strongly discouraged since wine wastes affect the soil erosion and decrease the groundwater quality because of the high organic matter losses [30,31]. As animal feed, grape pomace represents a problem because of its high amount of polyphenols that bond with proteins, leading to compounds not suitable for nutritional applications [24,32]. In addition, with the 2013, Reg. No 1308/2013—European Decree in the matter of wine wastes disposal rules—also distillation of wine by-products has stopped being a remunerative option for wine companies [19]. For these reasons, along with the necessity to invest in new sustainable and renewable materials, wine wastes have started to be investigated also within other different sectors. 

### 2.2. Composition and Trends of Use of Wine By-Products

#### 2.2.1. Vine Shoots

Vine shoots are non-wood lignocellulosic agricultural residues discarded during the vine pruning operations to equilibrate the growth of vegetation and fruit and to enhance the grape quality and quantity. With an approximate generation of 1.4–2 ton/ha per year [28], vine shoots represent the major amount of the wastes obtained in viticulture. The main components of vine shoots are cellulose (34%), hemicellulose (19%), and lignin (27%) [33], but also proteins, tannins, and ashes are present in minor quantities (Table 3). Vine shoots have been commonly destined to land-spreading or burned in fields. Recently, they have been investigated as source of antioxidant [48], bio-stimulating extracts [49], as woody flavorings [50], as absorber of toxic compounds [51], and as biomass for energy production [52]. 

#### 2.2.2. Grape Stalks

Grape stalks represent about 14% in weight of the total winemaking solid wastes and 3–5% of the processed grapes [19]. They are lignocellulosic materials with a reported composition of 17–26% lignin, 20–30% cellulose, 15–20% hemicelluloses, 6–9% ash, and a high presence of tannins (around 16%) [36,37,38]. These tannins are structurally associated with lignin and, therefore, the extraction of pure cellulose from grape stalks is not easy. Tannins represent 80% of the stalks’ phenolic compounds and their average degree of polymerization is of approximately 9 [53]. Most present polyphenols in stalks are flavanols (catechin, gallocatechin, and condensed tannins) as well as ellagitannins (hydrolysable tannins). The uses of grape stalks are similar to the uses previously described for vine shoots due to their similar compositions. Until recent years, grape stalks were generally land-spread, burned, or land-filled, while today researchers are valorizing grape stalks as absorbers of toxic compounds [54], as fermentable biomass for bio-ethanol production [55], as substrate for the preparation of activated carbons [56], and through the extractions of high-valued antioxidants [31].

#### 2.2.3. Grape Pomace

Grape pomace is the main fraction among the solid wine wastes derived from the winemaking processes steps. It represents up to 60% of the wine solid wastes and 20–25% of the processed grapes. They are formed by a mixture (generally 1:1 *w/w*, but it strongly depends on grape variety) of peels and seeds, and, in some cases, residual stalks can be also found [19]. If grape pomace is obtained by red winemaking processes, it is a fermented waste in which few phenolic compounds and sugars are present because they have been extracted and converted, respectively, during alcoholic fermentation. If obtained from white wines (or grape juices), grape pomace is unfermented and potentially richer in sugars and in polyphenols. This material is a complex substrate composed of 30% neutral polysaccharides, 20% acid pectic derivatives, 15% insoluble proanthocyanidins (condensed tannins), lignin, proteins, and other polyphenols. Polyphenols and proteins are often cross-linked to the lignin-carbohydrate fractions, and represent valuable organic sources [57]. As previously said, grape pomace has been almost exclusively destined to distillation steps (especially in the three top producer countries: Italy, France, and Spain). Nowadays, there is a great interest in the recovery of high-value products as polyphenols [58], colorant pigments [59], and organic acids such as maleic, citric, and tartaric [60]. On the other hand, because of their high quantity of sugars, grape pomace is also investigated as fermentative substrate [61,62,63] and as substrate for pullulan polymer production [64].

#### 2.2.4. Grape Peels

Grape peels (or skins) represent nearly 50% in weight of the grape pomace, even if the ratio of peels and seeds can significantly vary depending on the grape variety. Grape skins contain proteins (5–12%), ash (2–8%), soluble sugars (from 1% up to 70%, strongly depending on the wine process used), and above all, of polyphenols and dietary fibers. In grape peels the total dietary fiber content is almost 60% of the dry matter, and nearly 98.5% of them are insoluble [41]. Grape peels are also well known for their important content of polyphenols. These natural compounds are present both within and outside the cell-wall. Cell-wall polyphenols are bound to cellulose and hemi-cellulose through hydrophobic interactions and hydrogen bonds and, therefore, they can be scarcely extracted [19]. On the other hand, non-cell-wall polyphenols (including the phenols present in the vacuoles and those associated with the nucleus), being not bounded, are more easily extractable [42]. From a qualitative point of view, these polyphenols are characterized by many condensed tannins with high polymerization degrees (average value of 28 and maximum value of 80 [19]) and low amounts of hydrolyzable tannins (nearly 5%). In addition, not negligible amounts of anthocyanins such as delphinidin, cyanidin, and malvidin are findable within wine peels [19]. Finally, regarding the new techniques of valorization, it is possible to state that they are the same (or very similar) to the ones previously described for the grape pomace. 

#### 2.2.5. Grape Seeds

Grape seeds, the other fraction of grape pomace, has typically high contents of fibers (48%), proteins (11.5%), and lipids (13–15%) [43,44,45,65]. Wine seed oil generally contains high tocols and unsaturated fatty acids such as linoleic and oleic acids [66]. Within wine seeds are also present important amounts of polyphenols that are generally formed by the same constitutive units of peels’ polyphenols (catechins and gallocatechins) but that have lower polymerization degrees since they tend to be in monomeric form. On the other hand, the content of gallic acid derived polyphenols (hydrolyzable tannins) are more than 30% higher than those present within the skins and the stalks [42]. From an applicative point of view, wine seeds have been generally exploited for the oil recovery, that is used as food ingredient, and in other cases they have been directly burned in land to avoid expensive transportation costs. Nowadays, research is looking at wine seeds as an interesting source of antioxidants for the food, pharmaceutical, and cosmetic fields [67,68,69], as raw material for the fabrication of green graphene [70] and/or as a source of proteins [19].

#### 2.2.6. Wine Lees

Wine lees are defined as the residue formed at the bottom of recipients containing wine, after fermentation, during storage, or after other allowed treatments, as well as the residue obtained by filtration or centrifugation of this product [19,71] (Council Regulation (EEC) No. 337/79 [72]). They represent 2–6% of the processed grapes (in some cases up to 7.5% [73]) and, depending on their particle size and on the number of raking processes, wine lees can be classified as heavy and light lees. In each case, wine lees are composed by a solid fraction containing microorganisms (yeast and bacteria), insoluble carbohydrates (from cellulosic and hemi-cellulosic fractions), phenolic compounds, lignin, proteins, organic salts (mainly tartrates) [19], and by a liquid phase formed by ethanol (4–6%) and organic acids (tartaric, lactic, and acetic). Since they can be generated also during the clarification and filtration treatments, significant inorganic fractions can also be found within wine lees, since bentonite and kaolin are usually used in those winemaking processes. Wine lees have been generally used as feedstock for alcohol distillation and for tartaric acid recovery [74]. Nevertheless, recent wine lees applications have involved their antioxidant extraction [47], their use in food as ice-cream ingredients [75], and their exploitation as biochar for metals absorption [76].

## 3. Wine By-Products as Substrate for Polymer Synthesis 

### 3.1. Background

**Figure 2:**
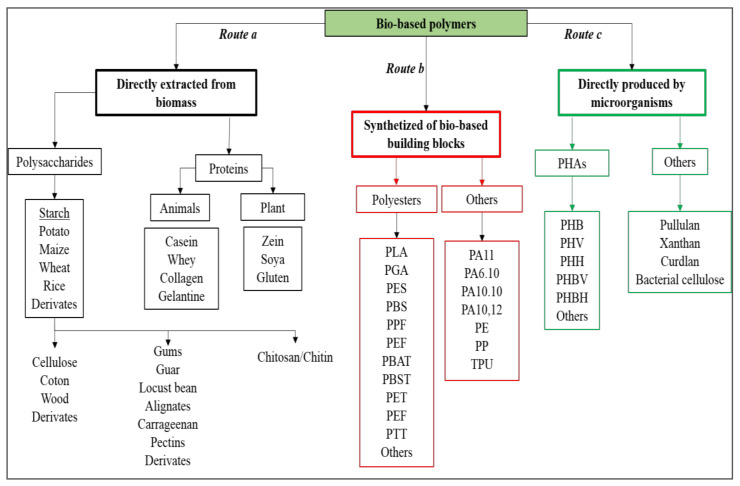
Bio-based polymers classification: (a) directly extracted from biomass, (b) obtained through polymerization of bio-based building blocks, and (c) directly produced by microorganisms. PLA = poly(lactic acid); PGA = poly(glycolic acid); PES = poly(sulfone)s; PBS = poly(butylene succinate); PPF = poly(propylene 2,5-furandicarboxylate); PEF = poly(ethylene 2,5-furandicarboxylate); PBAT = poly(butylene adipate-co-terephthalate); PBST = poly(butylene succinate-co-terephthalate); PET = poly(ethylene terephthalate); PTT = Poly(trimethylene terephthalate); PA11 = polyamide 11; PA6.10 = polyamide 6.10; PA10.10 = polyamide 10.10; PA10.12 = polyamide 10.12; PE = polyethylene; PP = polypropylene; TPU = thermoplastic polyurethane; PHB = poly(3-hydroxybutyrate); PHV = poly(3-hydroxyvalerate); PHH = poly(3-hydroxyhexanoate); PHBV = poly(3-hydroxybutyrate-*co*-3-hydroxyvalerate); PHBH = poly(3-hydroxybutyrate-*co*-3-hydroxyhexanoate).

As reported in Figure 2, bio-based polymers can be obtained by direct extraction from biomass (route a), by polymerization of building blocks partially or fully derived from natural feedstocks (route b), and/or by direct microbial synthesis (route c). From a technological point of view, bio-based polymers directly extractable from biomass (e.g., starch or cellulose, route a in Figure 2) are generally blended with other polymers because their intrinsic properties and their processability are not good enough for their direct use. On the other hand, the obtainment of bio-based building blocks from renewable feedstocks (route b in Figure 2) is increasingly gaining importance since they can lead to sustainable polymers or they could be converted into other important bio-based chemicals, in perfect agreement with the bio-refinery concepts. Similarly, the direct synthesis of polymers from microorganisms (route c in Figure 2) is also very attractive because the obtained polymers (e.g., PHAs) have interesting and versatile properties. 

**Figure 3:**
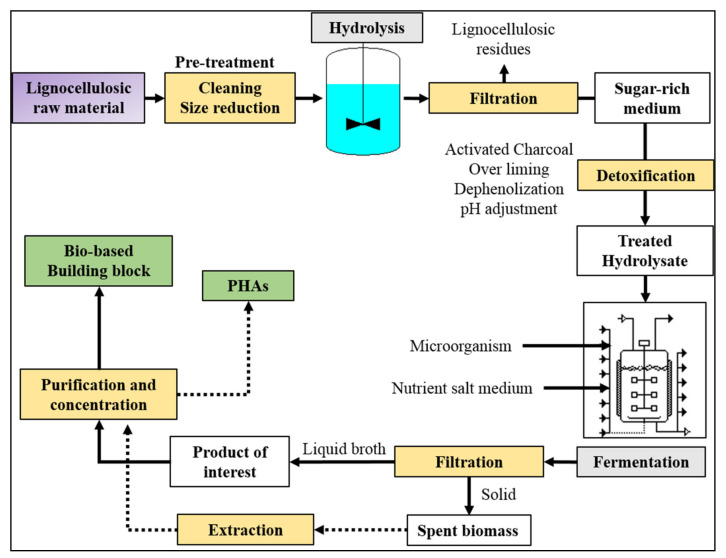
Schematic diagram for the obtainment of bio-based building blocks and poly(hydroxyalkanoates) (PHAs) using lignocellulosic raw materials.

In the case of routes b and c, the starting renewable raw materials generally are starchy, sugary, and/or oily feedstocks (first generation) or lignocellulosic materials (second generation). In each case, the first processing step involves a sugar extraction from the starting raw material. Sugary first generation crops such as sugar cane and beet juices do not need particular pre-treatments since they directly release simple sugars. Starchy materials, which are formed by two different polysaccharides: amylose, a long linear glucose polymer with few branched chains and amylopectin, a short and highly branched polymer of the α-glucose. They are generally liquefied by thermostable α-amylase and subsequently saccharified by α-amylase and amyl-glucosidase to avoid starch gelatinization [77]. Being formed by long chains of glucose (cellulose) and by long chains of hexoses and pentose sugars (hemicellulose), lignocellulosic materials need to be pre-treated to breakdown the lignocellulosic structure and subsequently hydrolyzed (by acid, basic, or enzymatic treatment) to release simple sugars. Successively, obtained sugar medium can be converted to useful products or building block precursor through reactions of oxidation, dehydration, hydrolysis, hydrogenation, and catalysis, or via fermentation. The most important polymer precursors such as ethanol, lactic acid, succinic acid, and PHAs are obtained through microbial synthesis (Figure 3). 

Using lignocellulosic materials as starting feedstock, sugar hydrolysate medium is generally detoxified before fermentation by one or more treatments such as activated charcoals, overliming, dephenolization, and delignification [78,79,80,81]. Indeed, products such as furfural and 5-hydroxymetyl furfural (5-MF), organic acids, polyphenols, and lignin are generally released during hydrolysis, and being microorganism inhibitors, they have to be removed before fermentation. Nevertheless, in some cases, the sugar degradation is a wanted process; as an example, 5-HMF can be converted into 2,5-furandicarboxylic acid (FDCA) [82], or levulinic acid [83] which can be successively transformed in succinic acid [84]. Therefore, adopted treatments are a function of the initial raw material typology (starchy, sugary, or lignocellulosic) as well as of the desired final product. 

### 3.2. Ethanol

As previously reported in Figure 1, after alcoholic fermentation grape pomace and wine lees are removed from wine. These by-products contain important amounts of ethanol (4–6% wt.) [19] which is generally recovered by distillation. In addition to conventional distillation, ethanol could be obtained from wine wastes also through fermentation of suitable hydrolysates. This route seems particularly efficient in the case of lignocellulosic wine wastes, such as grape stalks [55] and vine shoots [85], or in the case of grape pomace still rich in fermentable sugars [86,87,88] (e.g., white grape pomace that have been not fermented). While bio-ethanol obtained from wine wastes is usually used for food, pharmaceutical, and cosmetic sectors or used as fuel for energy and transport purposes, it can be destined also to polymer applications. Indeed, through catalytic dehydration, bio-ethanol can be easily converted into ethylene for the production of bio-polyethylene (bio-PE), one of the most large-scale produced bio-based polymers (200 kton in 2018). Bio-based ethylene could be reacted with chlorine to obtain 1,2-dichloroethane which can be further transformed by dehydrochlorination in vinyl chloride, the starting monomer of polyvinylchloride (PVC) [89]. Similarly, bio-ethylene could be reacted with acetic acid (also obtainable from bio-ethanol through oxidation) and oxygen to form vinyl acetate that could be successively polymerized to give bio-based polyvinyl acetate (PVA) and/or poly(vinyl alcohol) (PVOH) [89]. It is also possible to produce bio-based ethylene glycol by catalytic dehydration of bio-ethanol for the production of bio-poly(ethylene terephthalate) (PET) and this route of conversion is nowadays conduced at a large scale for the obtainment of bio-based bottles [90]. Moreover, some studies have shown the possibility to exploit grape pomace for the fermentation into butanol and acetone (ABE fermentation) [91] and therefore further bio-based building blocks can be derived from wine wastes. Reduction of acetone to isopropanol, followed by reduction to propylene could lead to bio-based polypropylene (PP), while isobutanol could be exploited as a building block for polyesters and synthetic rubbers. In addition, bio-based isobutanol can be dehydrated into isobutylene which can be further converted by dimerization and de-cyclization to *p*-xylene in high overall yields (Gevo Inc. and Toray Industries process [92,93]). Therefore, *p*-xylene can then be oxidized to form bio-based terephthalic acid in order to potentially obtain fully bio-based PET. 

### 3.3. Lactic Acid

Poly(lactic acid) (PLA) is one of the most produced biopolymers (218 Kt in 2018) and it is a biodegradable linear aliphatic polyester industrially obtained by ring opening polymerization (ROP) of lactide, the lactone cyclic di-ester derived from lactic acid (LA). Today LA is totally produced by bacterial fermentation, PLA is a fully bio-based polymer [94]. Lactic acid exists in two different optically actives forms: *L*- and *D*-lactic acid and consequently different forms of lactide can be obtained depending on the starting lactic acid molecules. In particular, it exists as the *L*-lactide (formed by two L-lactic acid molecules), the *D*-lactide (formed by two D-lactic acid molecules), the *meso*-lactide (formed by one *L*- and one *D*-lactic acid molecule), and the raceme lactide (formed by an equimolar mixture of *L*- and *D*-lactides). During polymerization, the chirality of the lactide monomer can modulate the polymer stereochemistry leading to several different PLA forms such as PLLA, PDLA, or PDLLA, which are tremendously different in terms of mechanical properties. As an example, PLLA, which is the most commercialized resin, is a semi-crystalline, hard, and brittle polymer having a melting temperature of 165–185 °C and a glass transition temperature of around 55–65 °C, while atactic PDLLA is a completely amorphous material with a significantly lower tensile strength. Crystallinity is also strongly affected by the *L*-lactic acid content and PLA resins with more than 93% *L*-lactic acid are mainly semi-crystalline, while with 85%, PLA is amorphous [95]. The bio-fermentation of lactic acid has been favored by the fact that pure *L*-lactic acid cannot be easily obtained through chemical synthesis, while, in nature, the *L*-lactic acid form is the most abundant one and many microorganisms can synthetize it. At industrial scale, homo-fermentative microorganisms are preferred to hetero-fermentative since they ensure higher yields and less by-products (hetero-fermentative microorganisms produce just 1.8 mole of LA per mole of sugars and many by-products such as acetic acid, glycerol, and carbon dioxide) [96]. Typical homo-fermentative microorganisms used for LA production belong to the *Lactobacillus* family and most of them synthetize *L*-lactic acid (such as *L*. *amylophilus*, *L. bavaricus*, *L. casei*, and *L. maltaromicus*) but *D*-lactic acid or *D*- and *L*- mixtures can be also obtained using *L. delbrueckii*, *L. jensenii*, and *L. acidophilus* [96,97]. Today, LA is currently produced in large scale exploiting sugar or starch-rich biomasses such as sugarcane and maize, but several companies and researchers are investigating the use of lignocellulosic raw materials, to decrease final PLA price and to avoid food competitiveness concerns. In this perspective, lignocellulosic vine shoots represent a very promising substrate for lactic acid production (Table 4). Bustos et al. [98] investigated vine shoots hydrolysate (18 g/L of xylose, 11 g/L of glucose, and 4.3 g/L of arabinose) as carbon substrate for the LA production by *Lactobacillus pentosus* obtaining a final LA concentration of 21.8 g/L. Moreover, the same research group [99], in combination with the vine shoot hydrolysate as carbon source, employed distilled white wine lees (20 g/L) as a source of nutrients to further reduce final LA costs, achieving a promising volumetric LA productivity of 3.10 g/Lh and a conversion yield of 70% (g of LA per g of sugars). Indeed, the nutrients used traditionally in most fermentative media (particularly yeast extracts and peptone) are very expensive and they account for almost 30% of the total cost of the process [100], and wine lees, being formed by dead yeasts, could represent a potential and cheap source of nutrients. For this reason, many authors have tested red and/or white lees (before and/or after ethanol or tartaric acid recovery) as fermentative rich in nutrients broth [101,102,103]. In particular, Bustos et al. [103], using 20 g/L of wine lees coming from the second decanting step (before distillation) as unique nutrient and *Lactobacillus rhamnosus* as microorganism, achieved an excellent LA production of 105.5 g/L and a volumetric productivity of 2.470 g/Lh. Finally, some authors have exploited wine by-products as fermentative carbon source to simultaneously obtain LA and other high-value products during the same process. As an example, Rivas et al. [104] employed hemi-cellulosic hydrolysates from vine shoot trimmings as carbon source for a two-stage bioreactor. In the first step, they produced LA converting glucose by *Lactobacillus rhamnosus* (31.5 g/L of LA) and in the second stage, they fermented the xylose into xylitol by *Debaryomyces hansenii* after *L. rhamnosus* removal trough microfiltration. Similarly, Rodríguez-Pazo et al. [105] exploited vine shoot hydrolysate to obtain, in addition to LA, also phenyllactic acid and biosurfactants by implementing a simultaneous saccharification and fermentation (SSF) carried out by co-cultures of *L. plantarum* and *L. pentosus*. 

### 3.4. Poly(hydroxylalkanoates) (PHAs)

Poly(hydroxyalkanoates) (PHAs) belong to a family of fully biodegradable polyesters that generally consist of 3-, 4-, 5-, and 6-hydroxyl-carboxylic acids. PHAs are usually accumulated by bacterial fermentation [108] or, less often, by transgenic microorganism [109] and plants [110] in intracellular granulates as carbon and energy reserves when nutrient supplies are imbalanced. PHAs are very interesting biopolymers because of their renewability, biodegradability, biocompatibility, and extreme versatility. The versatility of PHAs depends on the number of carbon atoms in PHAs constituent monomers. Depending on synthesis pathway, processing conditions, used substrate, and bacterial culture more than 150 kinds of hydroxyl-carboxylic acids may be obtained as PHA monomers [111] and carbon number may vary from 3 to 16. This aspect gives PHAs an extended spectrum of associated properties, which may make PHAs similar both to thermoplastics polyolefins (short-chain length, scl-PHAs, 3–5 carbon units) and to elastomers and adhesives (medium-chain length, mcl-PHAs, 6–14 carbon units) [112]. For these reasons, it is believed that PHAs could be able to replace the 33% of common commercial petroleum polymers [113,114]. Despite this potential, PHAs are scarcely present in the market because of their high costs which are generally 3–5 times higher than those of conventional petroleum polymers [13]. Today there are three crucial factors in PHAs production that need to be enhanced and/or optimized to decrease the PHAs’ final prices. Firstly, it is of fundamental importance to develop new systems for the recovery and purification of PHAs [115]; indeed, until today, PHAs are generally extracted using chloroform, which is toxic, not eco-friendly, and expensive from an energetic point of view (it needs to boil) [116]. The most promising alternatives regard the mechanical cell disruption, the chemical or enzymatic cell mass digestion, the osmotic extraction [117], the use of green solvents such as lactic acid esters [118], and/or the use of solvents derived by the fermentation of the same substrate used for the PHAs synthesis. For example, the Brazilian company PHBISA uses sugar canes both as poly(3-hydroxybutyrrate) (PHB) substrate and as substrate for the fermentation of iso-pentanol which is subsequently used as PHB extractive solvent [116,119]. Several efforts are in progress in order to decrease the PHAs’ costs optimizing the fermentative processes. In fact, today, PHB synthesis is generally carried out under discontinuous conditions (batch bioreactors) which are characterized by low PHA yields and/or productivities. Different strategies such as cell-recycle or the use of continuous fermenters could lead to the obtainment of higher yields and to a significant decrease of the processing costs [120]. In particular, one of the most promising configurations is the utilization of multi-stage bioreactor cascades [121]. Following this approach, several (from two to five) bioreactors are disposed in series to simulate tubular continuous reactor conditions and to optimize each PHA production phase. Microorganism growth is conducted within the first reactor, under ideal conditions of cultivation; meanwhile PHAs accumulation is carried out in the other following reactors optimized in accordance with the microorganism kinetics. Finally, PHA price can be significantly lowered by the use of inexpensive and non-food competitive raw materials such as sugar substrate for the microorganisms [16]. In particular, it is reported that the involved substrate plays a cost-effective key role, affecting up to 38–50% the final cost of the PHAs [122,123,124]. Therefore, there is a growing interest in using wine by-products as raw material for the PHAs production (Table 5); indeed, being cheap and abundant, they could significantly decrease the final PHA cost. Follonier et al. [125] investigated the possibility of using Solaris grape pomace as carbon source for the production of mcl-PHAs. They enzymatically converted residual polysaccharides of the grape pomaces into fermentable monosaccharides (106 g/L) and, in a 0.1 L bio-fermenter, they exploited *Pseudomonas resinovorans* microorganism to achieve a PHA production of 21.3 g/L and a productivity of 0.05 g/Lh. The same authors [126] obtained higher volumetric productivity (0.10 g/Lh) by employing *Pseudomonas putida* KT2400 as microorganisms, sugars extracted from Gewurztraminer pomaces as carbon substrate, and a 100L bioreactor operating in fed-batch conditions as apparatus. Martinez et al. [127] developed a multi-purpose four step-cascading bio-refinery aimed to valorize the grape pomaces from red grapes *Vitis vinifera L*. varieties. In particular, they used dephenolized grape pomace to produce volatile fatty acids (VFAs) by anaerobic acidogenic digestion and they exploited these VFAs as carbon substrate for the production of mcl-PHAs. Kovalcic et al. [128] used oils and fermentable sugars derived from grape pomace as carbon nutrient for *Cupriavidus necator* to produce PHB in a 2-L reactor, obtaining a volumetric PHB productivity of 1.363 g/Lh. Moreover, in addition to fermentable sugars and lipids, authors also isolated pigments, phenolic compounds, lignin, and cellulose from the same wine peels and seeds, proposing a circular system of bio-refinery. Similarly, Dimou et al. [46] developed a novel wine lees-based bio-refinery able to produce antioxidants, tartrate, ethanol, and PHB. In contrast to previous reported works, Dimou et al. exploited wine lees as nutrient-rich supplement medium, with the aim to substitute commercial yeast extracts, and not as carbon source, that was in fact crude glycerol. In particular, they exploited solid and liquid fractions of wine lees to produce a nutrient-rich supplement as substitute for commercial yeast extracts and they used crude glycerol as carbon source. 

### 3.5. Succinic Acid

**Figure 4:**
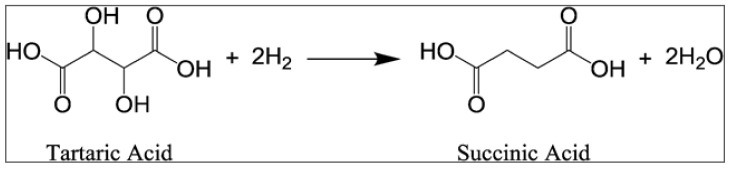
Direct conversion of tartaric acid in succinic acid.

Succinic acid (SA) is a four-carbon dicarboxylic acid recognized as one of the most promising biomass derived chemicals [129]. This versatile C4 building block can be used for different applications such as food, pharmaceutical, and chemical, or can be exploited to replace maleic anhydride to produce valuable products such as tetrahydrofuran, 1,4-butanediol, plasticizers, and biodegradable polymers such as poly(butylene succinate) (PBS) and poly(butylene succinate–*co*-adipate) (PBSA) [130]. Today, SA is generally produced through several reactions starting from petrochemical products (e.g., oxygenation of butane to maleic anhydride, which is successively hydrated to form maleic acid, which finally yields SA through hydrogenation). Since this process is not carbon neutral, in recent years several attempts to obtain bio-based SA through fermentation of agro-industrial derived sugars have been carried out [131,132,133]. Nevertheless, the main drawback is that, following this strategy, low SA concentrations and high concentrations of organic acid by-products (e.g., acetic, formic, and pyruvic) are generally obtained [134,135,136]. An alternative and efficient strategy could be the direct conversion of tartaric acid in succinic acid (Figure 4). Tartaric acid (TA) is structurally similar to succinic acid (except for the two OH groups at C_2_ and C_3_ positions) and largely available in wine lees (100–150 kg/ton) and grape pomace (50–75 kg/ton) [73], from which it is generally recovered via adsorption, extraction, ion exchange, and/or electro-dialysis [137]. Li and Zhang [138], using a two-steps process characterized by deoxydehydration reaction (DODH) of TA in maleic acid intermediate (91% yield) using NH_4_ReO_4_ catalyst in 3-pentanol, followed by hydrogenation in water of maleic acid using Pt/CC catalysts, have been able to synthetize SA from TA with a promising overall yield of conversion of 86%. Similarly, Fu and co-workers [139] have developed a novel one-step process for catalytic and selective production of SA from TA using a liquid-phase system comprising a molybdenum oxide catalyst supported on carbon black (MoO_x_/BC) and hydrobromic acid in acetic acid. Under optimized conditions of reactions (T = 170 °C, P = 37 bar, and reaction time of 24 h), they reached a yield of 86% of SA. 

## 4. Wine By-Products as Reinforcing Fillers

### 4.1. Background

Polymer composites are materials of great importance because of their ability to provide unique properties that would not exist naturally. Moreover, the final properties of polymer composites can be tailored depending on the selective design composition, the processing apparatuses, and the final application. Polymer composite science is an age-old study that dates back to 1910, when glass fibers were first exploited within synthetic plastics [140]. Similarly, bio-fibers were first introduced in 1941, when Henry Ford introduced biocomposites using hemp, sisal, and cellulose as natural reinforcing fibers [141]. Since then, biocomposites have gained more and more importance within both industry and academia as testified by the number of publications regarding biocomposites which has increased from 50 in 2005 to 250 in 2013, and by the production volumes of fillers which has increased from 525 kton in 1967 to 3.9 Mt in 2010, just in the United States [142]. Polymer biocomposites consist of two or more distinct components, which, once mixed, produce a new material with very different properties from those of the individual components. Generally, with the term biocomposite are intended all materials formed by natural fillers/fibers (NFs) mixed within a polymer/biopolymer matrix and/or synthetic fillers mixed within a biopolymer matrix and clearly, from an ecological point of view, NFs included within biopolymers represent the greenest solution. If compared with synthetic fillers, NFs have higher toughness, lower density, and are less abrasive for the processing equipment. In addition, NFs ensure lower pollutant emissions, and they can reduce the carbon footprint of not-fully bio-based biopolymers [143]. NFs represent also a cost-advantage alternative to conventional fibers, which are expensive and require important energetic efforts to be produced. For example, glass fibers are priced 1.2–1.8 €/kg and they require 30 GJ/ton of energy, while carbon fibers cost 12–15 €/kg and need around 130 GJ/ton [144]. The use of wine by-products as reinforcing fillers within biopolymer is a very intriguing route since wine wastes are cheap (e.g., wine lees costs just 0.045 €/kg and grape pomace 0.022 €/kg [145]), abundant (especially in Europe) and, as it will be shown in next paragraphs, they can be processed up to relatively high temperatures without significant mass losses. Thanks to these three properties, wine by-products are very promising for large-scale applications. 

### 4.2. Wine Derived Fillers: Physical Properties 

All the works reported in the literature regarding biocomposites formed by biodegradable (PLA, PBS, and scl-PHAs) and/or bio-based (PA11) polymers and wine by-products as reinforcing fillers have been listed in Table 6 and discussed in further paragraphs. 

In these papers, wine by-products have been generally oven-dried and ground into a fine powder and subsequently mixed in percentages ranging from 5 to 50 %wt. within biopolymers by the means of twin-screw extruders or internal mixers. The physical parameters as density, mean particle size, and moisture content have been compared in Table 5. It can be firstly noticed that there are no significant differences in terms of particle density (1.36–1.44 g/cm^3^). Nevertheless, from an applicative point of view it is useful to report that grape stalks and vine shoots, before grinding operations, have a very low apparent density (around 0.03 g/cm^3^) [154] that could affect the transport costs during large-scale operations. In terms of mean particle size, wine lees have exhibited the lower values (D_50_ of 25 μm) [146] and grape seeds the highest (D_50_ of 750 μm) [155,156]. These differences have been explained by the fact that grape seeds, containing important amounts of oleic and linoleic acids [157], tend to form aggregates, while wine lees, being formed by high inorganic fractions (40%), can be easily grinded into tiny particles. For lignocellulosic fillers (grape stalks and vine shoots) it is hard to define the aspect ratio and the particle size since they are formed by different size particles (from 5 mm to 300 μm) which are covered and tangled with long branched fibers [149]. The degradative temperatures (*T*_deg_) of the wine fillers, evaluated by thermogravimetric analysis (TGA) under nitrogen atmosphere, have been summarized in Table 7. Wine lees have exhibited the best thermal stability (*T*_deg_ of 267 °C), while grape peels and grape pomace have started to degrade already at 180–190 °C, probably because of the low stability of their components such as pectin and non-structural sugars. Finally, lignocellulosic grape stalks and vine shoots degrade in the 208–240 °C range and their thermal stability strongly depends on their hemicellulose (less stable), cellulose (intermediate), and lignin (most stable) composition [158]. In conclusion, TGA data confirms that wine fillers are stable until around 200 °C and that they can therefore melt compounded with polymers having a processing temperature below 200 °C.

### 4.3. Effect of Fillers on Mechanical Properties 

**Figure 5:**
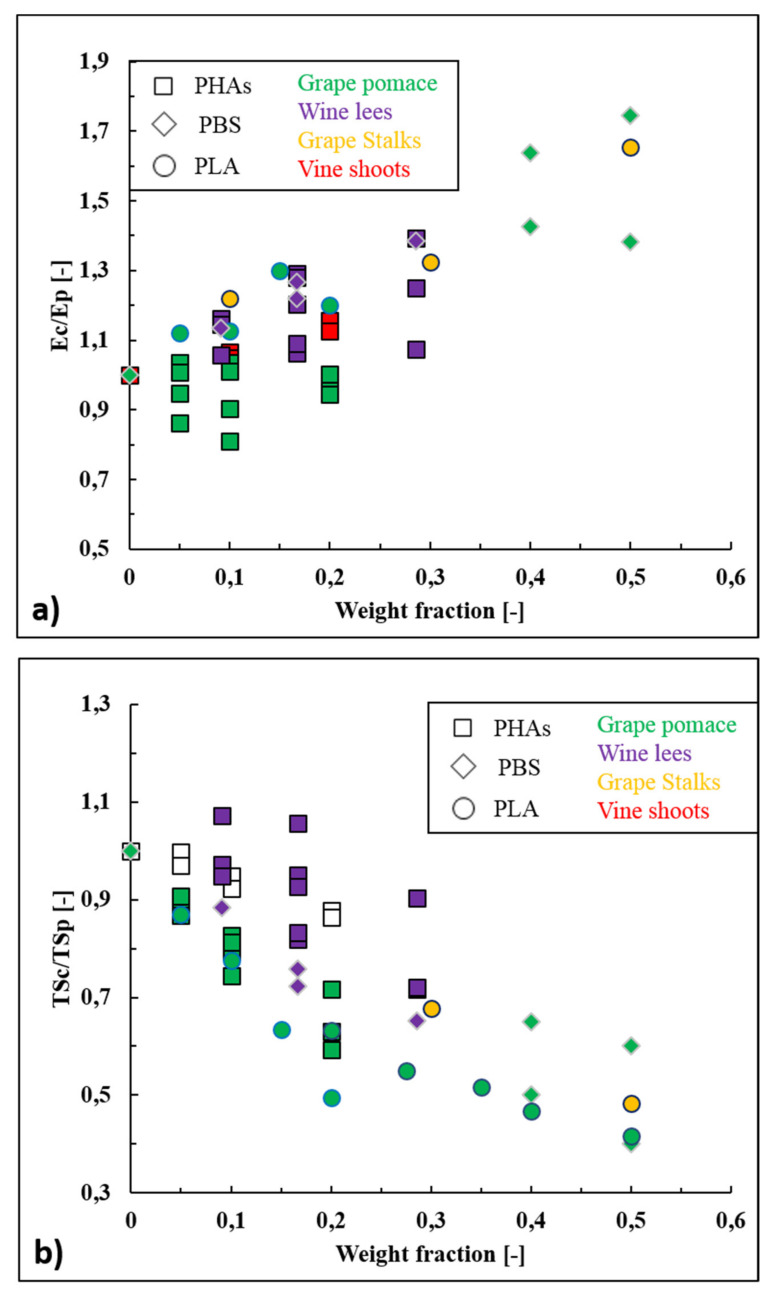
(**a**) Ratios (*E*_C_/*E*_P_) between Young’s modulus of wine-based composites (*E*_C_) and neat biopolymers (*E*_P_), and (**b**) ratios (TS_C_/TS_P_) between tensile strength of wine-based composites (TS_C_) and neat biopolymer (TS_P_) as function of the wine filler content.

The data reported within works listed in Table 6 have been analyzed and the ratios (*E*_C_/*E*_P_) between Young’s modulus of wine-based composites (*E*_C_) and the neat biopolymers (*E*_P_) have been plotted as function of the filler loading in Figure 5a. From an overall point of view, it can be noticed that elastic modulus increases almost linearly with the wine filler content. The gain in Young’s modulus has been explained by the fact that wine fillers have higher intrinsic stiffness than those of the biopolymer matrices. In terms of wine filler typology, it is difficult to make a precise comparison since mechanical properties of natural fillers depend on their chemical composition, which changes according to the grape variety. In general, the amount of cellulose and hemi-cellulose are the most affecting parameters since cellulose (140 GPa) is much stiffer than hemicellulose (8 GPa) [159]. For example, Battegazore et al. [149] reported the ability of grape stalks, which have high amounts of cellulose (20–30 %wt. [36]), to enhance up to 65% the elastic modulus of PLA, and, exploiting the micro-mechanical models of Voigt and Halpin–Tsai, they also calculated that grape stalks had an intrinsic elastic modulus (*E*_f_) of 6.8–9.0 GPa. David et al. [34] tested both vine shoots and grape pomace as reinforcing fillers within PHBV and they noticed that vine shoots were able to increase the Young’s modulus (up to 16%) while grape pomace did not affect this property. Indeed, grape pomace is poor in cellulose (10%) and hemicellulose (6%) and particularly rich in lignin (35–42%) which, contrary to cellulose and hemicellulose, acts more as a coupling agent (between cellulose and hemicellulose) rather than as a stiffening element [160]. Finally, Nanni et al. [146,152] reported the wine lees’ ability to increase the elastic modulus of several different biopolymers such as PBS, PHBH, and PHBV, and they calculated an intrinsic elastic modulus of wine lees of around 4.2–7.3 GPa. The stiffening effect was explained by the presence of rigid inorganic particles such as potassium tartrates and alumina silicates within wine lees and by their low diameter size, which has favored the particle dispersion and homogeneity. 

The data reported in Figure 5b show that tensile strength has decreased almost linearly with the wine filler content for each considered biopolymer (PLA, PHAs, and PBS). Tensile strength is strongly affected by the particle-matrix adhesion, since poorly bonded particles cannot transfer the mechanical stress through the interface. The discontinuities and de-wetting phenomena that are usually generated in poor bonded systems lead to the formations of crazes for applied stresses lower than those observed with well-bonded particles [161]. Therefore, the decrease in tensile strength values can be mainly explained by the different polarity between biopolymers (slightly hydrophilic) and bio-fillers (highly hydrophilic). Physical treatments (e.g., plasma, corona, steam explosion, autoclave treatments) and/or chemical treatments (e.g., silanization, alkalization, acetylation, enzymatic treatments) of wine fillers should be carried out to enhance this mechanical property. As an example, plasma treatment has been adopted to enhance the tensile strength of biocomposites formed by PHB and flax fibers [162], PHBV and wood flour [163], PLA and jute fibers [164], and PLA and silk fibers [165] and silanization has been exploited to improve the mechanical resistance of biocomposites such as PLA and coconut shells [166], PBS and cotton fibers [166], and PHBH and cellulose [167]. Even if no treatment of wine fillers is reported in the literature, some authors have investigated the possibility to enhance tensile strength values of wine-based composites adding coupling agents as silane or maleic anhydride grafted polymers (Polymer-gMA) directly during the extrusion step (reactive extrusion). For example, Gowman et al. [153] increased the tensile strength values of composites formed by PBS and 40 %wt. (PBS40GP, TS of 20MPa) and 50% of grape pomace (PBS50GP, TS of 16 MPa) of 30% and 50%, respectively, adding 3–4% of PBS-gMA during the compounding step. Nanni et al. [146] conduced a reactive extrusion using PHBV as polymer matrix, 20 %wt. of wine lees as filler and 1 %wt. of 3-methacryloxypropyltrimethoxysilane as coupling agent, obtaining a biocomposite characterized by a TS value (34 MPa) even higher than one of neat PHBV (32 MPa). It can also be underlined that, in addition to polymer-particle adhesion, particle size and particle loading affect the tensile strength values of composites too. In particular, tensile strength tends to increase with decreasing particle size because higher particle specific areas (inversely proportional to the mean size) involves better stress transfer between the particle-polymer interfaces and this aspect explains why wine lees, being the finest tested powder, has guaranteed the minor tensile strength losses (Figure 5b). Similarly, the fact that tensile strength usually decreases with increasing the filler loading because of aggregation phenomena that deteriorate the stress efficiency [168], would explain why wine-based composites have exhibited lower TS values when used in the 30–50 %wt. range. From Figure 5b it is also observable that biocomposites filled with grape pomace have generally exhibited the lower TS values, if compared with the other wine by-products. This fact is partially unexpected because the high content of lignin (34–41%) and proteins (10–12%) within grape pomace should have guaranteed a better polymer-filler adhesion as a consequence of the ability of the backbones and side chains of these two macromolecules to form different interactions with the polymer at the molecular level [169]. A possible explanation of this behavior could be that grape pomace proteins, being already cross-linked with polyphenols, are slightly active and hindered to further interact with polymer chains. Moreover, the combined effect of high temperature and shear forces during processing steps such as extrusion and/or injection molding could have affected the proteins’ structure (e.g., formation of protein aggregations and protein denaturation) modifying their functionalities [170]. In each case, these hypotheses should be verified in further works aimed to underline the proteins’ role in grape pomace filled composites. As expected, elongation at break of biopolymers has been generally reduced by increasing the wine particles loading, as often reported in literature even for conventional composites [161,171]. This behavior of composites is due by the non-deformability of fillers that, indeed, create defects and promote crack propagation [161]. For example, the addition of 40 %wt. of grape pomace to flexible PBS has caused a dramatic loss of ductility (−94%) [153]. Nevertheless, PBS filled with 20 %wt. of fine wine lees powder (D_50_ of 25 μm) [152], exhibited an excellent elongation at break value (ε_b_ of 188%) pointing out that finely grinded wine fillers do not drastically affect this mechanical property. The great ductility of PBS and wine lees composites was also explained as consequence of a plasticization effect induced to PBS matrix by the lipid fraction (1.7 %wt., mainly linoleic and oleic acids) present within wine lees. Anyway, from an overall point of view, the potential plasticizing effect of the lipid fraction is generally hindered by the tendency of the filler itself to decrease the polymer ductility. As an example, polypropylene (PP) filled with just 6 %wt. of grape seeds, which are the wine by-products with the highest lipid content (10–15 %wt.), exhibited an elongation at break value of barely 13% (against 1000% of neat PP). Therefore, it can be stated that physical properties (e.g., mean particle size) overly prevail on the chemical composition (e.g., lipid content) in terms of final mechanical performance. To fully exploit the potential plasticizing effect of the wine by-products, lipid extracts should be used; nevertheless, being a food grade product, grape seed oil is generally tested for the fabrication of edible films [172,173] rather than as plasticizer for commercially available biopolymers. 

Finally, from a technological point of view, it can be also noticed that, since PLA and scl-PHAs are brittle materials with low elongation at break values, the addition of wine fillers would not affect the final suitable application fields of these biopolymers. 

### 4.4. Effect of Fillers on Other Properties 

#### 4.4.1. Flexural Properties and Impact Strength 

Flexural strength has decreased by approximately 40%, with respect to virgin biopolymer, in both biocomposites formed by PLA and grape stalks (30 and 50 %wt.) [149] and in those formed by PLA and grape pomace (20 %wt.) [153], while flexural modulus has increased by approximately 25%. This behavior can be explained by the same factors previously described for tensile strength and tensile modulus. On the other hand, Gowman et al. [153] reported that grape pomace (20 %wt.) increased the PLA impact strength by around 50% (from 20 to 30 J/m^2^), pointing out the ability of grape pomace fillers to act as energy absorption particles. Similarly, Saccani et al. [150] reported that impact strength of PLA was increased by the addition of 10–15 %wt. of grape pomace and successively decreased for higher grape pomace contents. 

#### 4.4.2. Thermal Properties

Melting temperature (T_m_) and crystallization temperature (T_c_) of biopolymers are not significantly affected by the addition of wine fillers. Battegazore et al. [149] reported the ability of grape stalks to act as nucleating agents within amorphous PLA, and with 50 %wt. loading the crystallinity was found to be 42%. A similar nucleating effect has also been found in composites formed by scl-PHAs (PHBH and PHBV) and wine lees for loadings up to 20% [147]. However, for higher loadings (approximately 30 %wt.), crystallinity was decreased by around 12–26% with respect to virgin PHAs, as a consequence of agglomeration phenomena. Thanks to their low mean diameter size, wine lees act as a nucleating agent (+18% crystallinity) even within high-crystalline PBS (X_C_ of 62%) [152]. Finally, wine derived fillers have not significantly affected the glass transition temperature (T_g_) of biopolymers. The addition of grape pomace to PBS [153] or of wine lees to PHBV [147] has increased T_g_ values of neat biopolymers of only a few degrees (1–4 °C), testifying that these fillers do not immobilize polymer chains along the particle-matrix interface. 

#### 4.4.3. Thermal Stability

Since degradation temperatures (*T*_deg_) of wine by-products are in the 200–270 °C range, while those of PLA, PBS, and PHB are at around, 330, 340, and 290 °C, respectively, the thermal stability of the resulting composites are generally lower thermal than ones of the neat biopolymers. For example, PLA and grape pomace composites start to degrade at around 255 °C (−75 °C with respect to pure PLA) [149] and PHBV filled by vine shoots or grape pomace has shown a *T*_deg_ at around 265–270 °C (−12°C with respect to neat PHBV) [34]. Considering PBS biopolymer, the addition of 40 %wt. of grape pomace has lowered the PBS thermal stability of around 79 °C (*T*_deg_ of 260 °C) [153] while the addition of almost 20 %wt. of wine lees has decreased the *T*_deg_ of 22 °C (*T*_deg_ of 318°C) [152]. On the other hand, Saccani et al. [150], investigating composites formed by PLA and grape pomace, reported a two-stage thermogravimetric behavior. Indeed, they have reported that, at lower temperatures, filled PLA samples were the first ones to degrade as a consequence of the loss of poorly thermally stable components (hemicellulose), but at higher temperatures, grape pomace was able to progressively shift TGA curves to higher temperatures values. This feature has also been found in other composites [174] and it has been mainly attributed to lignin, which is the most thermally stable component of lignocellulosic materials. 

From another point of view, wine by-products can be used also as thermal and UV stabilizers to postpone the polymer degradation. In fact, wine by-products are rich in polyphenols, a class of natural antioxidants, which, thanks to their structure (one or more aromatic rings with one or more hydroxyl group attached), can interrupt the polymer degradation. The possibility of using wine antioxidants (extracts) has been deeply investigated; wine by-product extracts have been tested as stabilizers within polyolefin [156,175,176], Mater-Bi [177], poly(3-hydroxybutyrate) (PHB) [147,178], and poly(butylene succinate) (PBS) [179]. Since antioxidant efficiency depends also on the polymer degradative mechanism, it can be summarized that simple polyphenol molecules (such as Gallic acid and simple Catechins) are the best stabilizing system for polyolefin, which are characterized by a radical degradative pathway, meanwhile long chain polyphenols (such as proanthocyanidins) are preferable within polyester, which exhibit a not radical chain-scission degradation. In fact, in case of radical degradation (such as PP and PE), simple polyphenols, being not sterically hindered, guarantee a higher number of collisions between phenolic hydroxyl groups and the polymer chains, and thus they result more reactive and efficient than polymerized polyphenols. On the other hand, when dealing with polyesters (not radical degradation), long-chain polyphenols can mitigate the molecular weight loss due to degradation, acting as chain extenders and cross-linking agents between the broken polyester chains. Therefore, from a qualitative point of view, polyphenols extracted from grape peels (polymerization degree of around 28) should be used within polyesters, meanwhile polyphenols extracted from grape seeds (polymerization degree of around 11) should be exploited to stabilize polyolefin.

#### 4.4.4. Thermo-Mechanical Properties 

With the upcoming ban of single-use plastics in Europe from 2021, biodegradable polymers are increasingly required for disposable packaging products such as cutleries, dishes, and cups. In these applications, materials need to be resistant to temperatures of hot foods and beverages (80–100 °C) without softening. For this reason, the ability of biofillers to enhance the thermo-mechanical properties of biopolymers is of particular interest. Thermo-mechanical properties of wine-based composites have been evaluated by different methods such as dynamic mechanical analysis (DMA), heat deflection temperature tests (HDT), and creep tests, and in each case, results have pointed out the wine fillers’ ability to enhance the heat resistance of neat biopolymers. For example, grape pomace (40 %wt.) has enhanced the PBS storage modulus for the whole range of tested temperatures (from −40 to 100 °C) and has increased the HDT temperature of around 9 °C [153]. Nanni et al. [152] investigated the effect of wine lees on storage modulus of several biopolymers and they proposed a formula suitable for the prediction of the storage modulus of wine lees-filled biocomposites ($E_{C}^{\prime }\left(T,V_{WL}\right)$). The equation, which fitted experimental data with a coefficient of determination R^2^ of 83.1%, is reported below

$$E_{C}^{\prime }\left(T,V_{WL}\right)=E_{M}^{\prime }\left(T\right)e^{1.24V_{WL}}$$

where $E_{M}^{\prime }\left(T\right)$ is the storage modulus of the neat biopolymer at temperature *T* and $V_{WL}$ is the wine lees’ volume fraction. In the same work, they also reported the ability of wine lees to enhance the PBS HDT value of approximately 12 °C. Similarly, grape stalks enhanced by 25–30% the storage modulus of amorphous PLA for temperature ranges below glass transition temperature (50–60 °C) [149]. However, above T_g_, storage modulus of composites drastically dropped to zero, underlining the predominant role of morphology to avoid softening behaviors. Finally, Nanni et al. [146,152] investigated the creep behavior at different temperatures (20, 40, 60, and 80 °C) of composites formed by several biopolymers and wine lees in different contents (10, 20, and 40 phr). Looking at their reported data, it can be observed that wine lees lowered the PHBH, PHBV, PBS, and PA11 creep compliance of around (in average) 38%, 57%, 23%, and 28% and that the use of silane as coupling agent further improved the creep resistance. In addition, they fitted creep data, with parameters models (e.g., Burgers and Kohlrausch-Williams-Watts (KWW) models) pointing out that PHBH- and PHBV-based composites deformed to creep slower and to a lower extent, while PBS- and PA11-based composites deformed to a lower extent but more rapidly with respect to neat biopolymers.

#### 4.4.5. Water Uptake and Permeability

David et al. [34] investigated the water vapor permeability (WVP) of composites formed by PHBV and vine shoots or grape pomace at different percentages (5, 10, and 20 %wt.). They reported that WVP of wine-based composites was significantly higher (up to five times) than that of neat PHBV and they mainly explained this result as a consequence of the high hydrophilic character of wine wastes. They noticed that the extraction of polyphenols from vine shoots and grape pomace increased the hydrophilic behavior of wine wastes and indeed the WVP values. Vine shoots-filled composites were more permeable than grape pomace ones, because cellulosic fractions (mainly present in vine shoots) are much more hydrophilic than lignin ones (mainly present in grape pomaces). Due to the increase in WVP, they suggested the use of PHBV and vine shoots composites (after polyphenol extraction) for applications such as horticulture and packaging of respiring products. Saccani et al. [150] studied the water uptake of composites formed by PLA and grape pomace and they noticed that the process of water uptake in composites was almost equal to one of the plain matrix and that the final amount of absorbed water was tending to a constant saturation value following a Fickian behavior. With 15 %wt. of grape pomace, they found a saturation value only 2.5 higher than of PLA, and since grape pomace was poor in hydrophilic hemicellulose, they related the increased water uptake to the increased porosity of composites. 

#### 4.4.6. Biodegradation 

Nanni et al. [146] carried out biodegradation test on biocomposites formed of wine lees and PHB both in soil and in marine water and they reported the ability of wine lees to enhance the biodegradation rate of PHB with an increase proportional to their content. In particular, PHB filled with 40 phr of wine lees biodegraded almost two times faster than neat PHB in both degradative environments. Generally, the increased biodegradability of biocomposite is due to the fact that natural fillers increase both the biopolymer hydrophilicity [180] and the biopolymer porosity [181] and thus adsorption and transport of water from surface to polymer bulk is favored. In addition to these factors, authors suggested that the impressive weight losses observed were due also to the high content of organic acids (e.g., tartaric, acetic, and malic) present within wine lees that would have accelerated the hydrolysis of the polymer chains [182]. In the same work, they also reported the ability of grape seeds-derived antioxidants to postpone the PHB biodegradation, pointing out the possibility to tune and control the biodegradation rate, balancing the amounts of seeds extracts (retarding effect) and wine lees (promoting effect). Similarly, Nanni et al. [179] reported that even small amounts of grape peel extracts were able to accelerate the in-soil biodegradation of PBS even if, after 180 days of burying, the final mass losses were very limited as a consequence of the high crystallinity of PBS that discouraged the microorganisms attacks [180,183]. Finally, David et al. [184] investigated the biodegradation of biocomposites formed by PHBV and vine shoots by respirometric tests in soil, also evaluating the effect of polyphenolic compounds. The incorporation of vine shoot fillers in PHBV slightly accelerated the overall biodegradation kinetics and all their produced biocomposites were considered fully biodegradable according to the European standard NF EN17033. Moreover, they reported a negative impact of polyphenols (which have excellent antimicrobial activities) on the biodegradation rate and, therefore, they proposed a bio-refinery concept in which vine shoots are used as cost-effective and biodegradation promoters within PHBV only after polyphenol recovery. 

## 5. Conclusions

The present work has reviewed the possibilities of using solid winemaking by-products, namely vine shoots, grape stalks, grape pomace (formed by grape seeds and grape peels), and wine lees, as renewable feedstocks for the production of bio-based polymers and as natural reinforcing fillers.

As renewable second-generation (not edible) feedstocks, wine by-products have been tested as carbon source for the microbial production of such important polymer precursors as bio-ethanol, lactic acid, and succinic acid, as well as for the direct synthesis of PHAs. 

**Figure 6:**
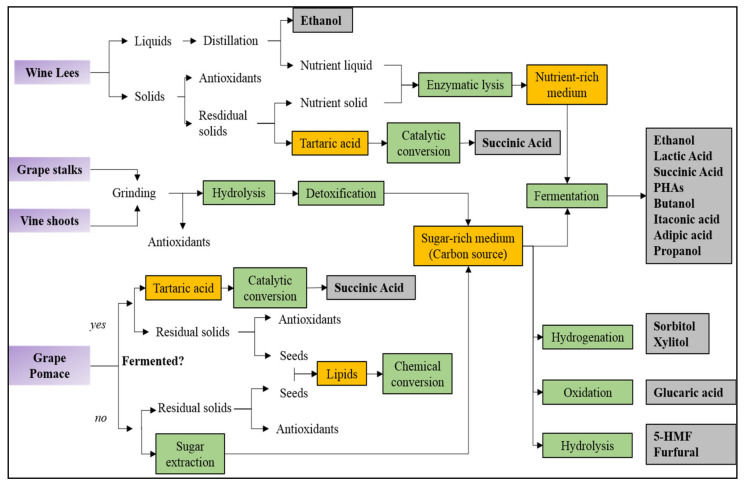
Proposed routes for the valorization of solid winemaking by-products as feedstocks for the fabrication of bio-based polymers.

Thanks to their lignocellulosic structure, vine shoots have been exploited as a natural carbon source for the microbial fermentation of bio-ethanol, lactic acid, and scl-PHA polymers with excellent results. The vine shoot hydrolysate, rich in C5 and C6 sugars, has guaranteed excellent yields of conversion and simple detoxification treatments have been enough to not inhibit the microorganism activity. As an example, the use of vine shoot hydrolysates as carbon source has led to lactic acid productions ranging from 15.5 to 43 g/L and considering that vine shoots are the most produced wine wastes (10.4–14.8 Mton for year), it can be concluded that they could be exploited with excellent results also at a large scale. Similarly, grape stalks represent a promising feedstock for the microbial production of polymer precursors. In particular, the grape stalk hydrolysate is particularly rich in C6 sugars, such as glucose, which are more easily metabolized by most microorganisms than C5 ones (more present in vine shoots). Nevertheless, despite their potentially higher yield of conversion, grape stalks have been tested as a carbon source less frequently than vine shoots because grape stalks also contain high amounts of antimicrobial tannins (16%) that, being bonded with structural lignin, are hardly removable. Therefore, future works should optimize the detoxification treatments to avoid the tannin presence within the grape stalk hydrolysate. From a technological point of view, both grape stalks and vine shoots have very light apparent densities and thus, they should be grinded in the field before their use to avoid expensive transport operations. The suitability of grape pomace strongly depends on the used winemaking processes. In particular, grape pomace generated after fermentation (e.g., red grape pomace) is not suggested for the microbial synthesis of polymers. In fact, simple sugars are present in low amounts since they have already been converted in ethanol by wine yeasts during wine fermentation and the hydrolysis of grape pomace would release only small amounts of sugars because cellulose and hemicellulose are present in small quantities (around 10–15%). Therefore, the best valorization route of this exhausted grape pomace is the recovery of tartaric acid and its further conversion in succinic acid. On the contrary, grape pomace not subjected to alcoholic fermentation (e.g., white grape pomace) could release simple sugars just by water extraction, without expensive and time-consuming hydrolysis treatments. Finally, wine lees, mainly formed by dead yeast and salts, have been successfully tested as nutrient medium and they could represent a cost-advantage alternative to commercial yeast extracts. In addition, tartaric acid generally extracted from wine lees could be converted in succinic acid for the production of bio-polyesters. These considerations have been schematized in Figure 6, where suitable routes to valorize each wine waste in new bio-based polymers have been proposed. 

From another side, wine wastes have been tested as reinforcing fillers within the most important biopolymers such as PLA, PHB, and PBS. Each wine waste has a degradative temperature higher than the processing temperatures of the mentioned biopolymers, but this property is strictly dependent on the chemical structure of wine wastes, which also depend on grape variety and on weather conditions. In addition, in large-scale, processing temperatures are generally higher than those adopted in lab scale. Therefore, the use of wine wastes as filler within PLA and PHB (high processing temperatures) is not always guaranteed and further works should test them as fillers also for biopolymers characterized by lower processing temperature, such as poly(butylene adipate-*co*-terephthalate) (PBAT) and polycaprolactone (PCL). In terms of mechanical properties, wine wastes have generally improved the stiffness of biopolymers and decreased their tensile strength. Grape stalks and vine shoots have enhanced the Young’s modulus of biopolymers thanks to their high contents of cellulose (which is much stiffer than hemicellulose and lignin), meanwhile wine lees have exhibited a positive stiffening effect because of their small diameter size and because of the presence of inorganic salts. In terms of tensile strength, wine wastes have generally decreased this mechanical property because of the poor adhesion between hydrophilic wine fillers and hydrophobic biopolymer matrix. Since in each work wine wastes have been used without any kind of treatment, it is reasonable to believe that surface treatments (e.g., silanization or acetylation) of wine fillers could improve this mechanical property; further works should verify this hypothesis. Other properties such as heat resistance, biodegradation rate, and vapor and/or water permeability have been increased by the use of wine fillers. In this case, further works should optimize the bio-composite formulation in order to improve biodegradability without significantly decreasing barrier properties. Finally, future research should test residual wine solids obtained from the synthesis of bio-based polymers as reinforcing fillers, also evaluating the possibility to fabricate fully wine-derived plastics formed by both wine-based polymers and fillers in perfect accordance with the circular economy’s principles. 

## Figures and Tables

**Table 1:** Bio-based content, biodegradability (yes/no), and production data (2018) [11,12] of the important biopolymers.

Biopolymer	Bio-Based Content [%]	Biodegradable*?	Production [kton]
Poly(lactic acid) (PLA)	100	yes	218
Poly(butylene succinate) (PBS)	50–100	yes	97
Poly(butylene adipate -*co*-terephtalate) (PBAT)	0	yes	152
Poly(hydroxyalkanoates) (PHAs)	100	yes	30
Thermoplastic starch blends (TPS)	25–100	yes	384
Polyethylene terephthalate (PET)	20–30	no	562
Polyethylene (PE)	100	no	201
Polyamides (PA)	40–100	no	245
Poly(trimethylene terephthalate) (PTT)	30–40	no	194

* The term biodegradable indicates a material that, thanks to its chemical structure, is able to undergo a process in which microorganisms such as bacteria and fungi metabolize the polymer chains into water, carbon dioxide (or methane), and biomass under aerobic (or anaerobic) conditions. From an applicative point of view, polymer products need to be certified as biodegradable under specific conditions depending on their expected end-life scenarios. As an example, plastic cutleries or bags, which are designed to be discarded into organic bins, need to meet the requirements of biodegradation described by EN13423 (industrial compostability), meanwhile, mulch films need to respond to the EN17033 (degradation on soil under weather conditions) or bioplastic items that could end up in the sea should be certified as biodegradable in marine environment (ASTM D6691). In these cases, the thickness of the bioplastic product and the biodegradation conditions (time, temperature, oxygen, and moisture levels) strongly affect the outcome of certification. In other words, the term biodegradable is a necessary condition for a biopolymer object to be certified but not a sufficient one.

**Table 2:** Production data of grapes, wine, and solid by-products in 2019.

	%wt ^a^	World [10^6^ ton]	Europe [10^6^ ton]	IT-FR-ES [10^6^ ton]	Italy [10^6^ ton]
Processed grapes ^b^	-	44.40	25.31	22.47	8.35
Produced wine	-	29.20	16.64	14.78	5.49
Vine shoots ^c^	1.4–2.0	10.36–14.80	4.76–6.80	3.45–4.93	0.99–1.41
Grape Stalks	3–5	1.33–2.22	0.74–1.25	0.67–1.12	0.25–0.42
Grape Pomace	20–25	8.88–11.10	5.06–6.33	4.49–5.62	1.67–2.09
Wine lees	2–6	0.88–2.66	0.50–1.52	0.45–1.35	0.17–0.50

^a^ %wt. is the weight fraction of by-product with respect to the processed grapes. ^b^ Considered the grapes used for wine production. ^c^ The generation rate of vine shoots is of 1.4–2 tons for each hectare of vineyard. This by-product is obtained also from grapes destined to table or spirits (not only for wine production). The global area under vines is 7.4 Mha, the European area is 3.43 Mha, the Italian, French, and Spanish area is 2.46 Mha, and the Italian one is 0.7 Mha.

**Table 3:** Wine by-products composition.

Wine by-product	Composition	Ref
Vine shoots	34% cellulose 20–30% hemicellulose 20–27% lignin 1.25% tannins 5% proteins 3–4% ashes	[28,33,34,35]
Grape Stalks	20–30% cellulose 15–20% hemicellulose 17–26% lignin 6–16% condensed tannins (insoluble) 6% proteins 6–9% ashes 1–3% soluble polyphenols	[36,37,38]
Grape Pomace	10.5% cellulose 6.1% hemicellulose 34–41% lignin 10% proteins 1–2% soluble polyphenols (strongly depends on if pomace is fermented or not) 8–9% ashes 15% condensed tannins (insoluble) 20% pectin substances 30% neutral polysaccharides	[34,39,40]
Grape Peels	5–12% proteins 2–8% ashes 2–5% lipids 1–2% soluble polyphenols (strongly depends on if pomace is fermented or not) 1–70% soluble sugars (strongly depends on if pomace is fermented or not) 60% dietary fibers - 98.5% insoluble dietary fibers - 1.5% soluble dietary fibers 4–6% pectin substances	[41,42]
Grape seeds	48% dietary fibers 11.5% proteins 13–15% lipids (oils and fatty acids) 5–8% polyphenolic compounds 60–70% carbohydrates	[43,44,45]
Wine lees	Solid phase: - Yeast and bacteria - Cellulose, hemi-cellulose and lignin - Proteins - Organic salts (tartrates) - Pectin substances (2–6%) - Pigments (1.2%) liquid phase: - Ethanol (4–6%) - Organic acids (tartaric, lactic, and acetic)	[19,46,47]

**Table 4:** Microbial synthesis of lactic acid using different wine by-products as fermentative source.

Substrate	Bacteria	Treatments	Fermentation Type	LA [g/L]	Y_LA_ [g/g]	P [g/Lh]	Ref
Vine shoots	*Lactobacillus pentosus*	Acid Hydrolysis and CaCO_3_ detoxification	Batch fermentation	21.8	0.77	0.844	[98]
Vine shoots and wine lees	*Lactobacillus pentosus*	Acid Hydrolysis and CaCO_3_ detoxification	Batch fermentation	15.5	0.70	3.1	[99]
Vine shoots	*Lactobacillus rhamnosus*	Acid Hydrolysis and CaCO_3_ detoxification	Two-stage sequential batch fermentation	31.5	0.93	1.312	[104]
Vine shoots	*Lactobacillus acidophilus*	Acid Hydrolysis and CaCO_3_ detoxification	Two-stage sequential batch fermentation	32.7	0.72	1.363	[106]
Vine shoots	*Lactobacillus pentosus*	Acid Hydrolysis and delignification	Saccharification and fermentation	43.0	0.68	0.253	[105]
Grape Pomace	*Lactobacillus pentosus*	Acid Hydrolysis	Batch fermentation	7.2	0.71	0.476	[107]
Wine lees	*Lactobacillus casei*	Alkaline hydrolysis assisted by microwaves	Batch fermentation	17.5	0.71	-	[101]
Wine lees	*Lactobacillus casei*	No treatments	Batch fermentation	65.8	0.82	-	[102]
Wine lees	*Lactobacillus rhamnosus*	No treatments	Batch fermentation	105.5	0.81	2.470	[103]

LA: Lactic acid production concentration (g of LA for liter of reactor); Y_LA_: Lactic acid yield with respect to the used carbon source; P: Lactic acid volumetric productivity.

**Table 5:** Microbial synthesis of PHAs using different wine by-products as fermentative source.

Substrate	Bacteria	Treatments	Fermentation type	PHA [g/L]	Y_PHA_ [%]	Y_sub_ [g/g]	P [g/Lh]	Ref
Grape pomace	*Pseudomonas resinovorans*	Enzymatic Hydrolysis	Batch fermentation	21.3	23.3	-	0.05	[125]
Grape pomace	*Pseudomonas KT2400*	Water Extraction	Fed-batch fermentation	21.8	77	-	0.10	[126]
Grape pomace	*Cupriavidus necator*	Dephenolization anaerobic digestion	Two fed-batch fermentations	-	68	0.27	-	[127]
Grape pomace	*Cupriavidus necator*	Enzymatic Hydrolysis	Batch fermentation	8.3	63	-	1.363	[128]
Wine lees		Enzymatic Hydrolysis	Fed-batch fermentation	30.1	71.3		0.56	[46]

PHA refers to the PHAs production concentration (g of PHAs for liter of medium); Y_PHA_ is the PHA content accumulated within the cell of microorganisms; Y_sub_ is the PHA yield with respect to the used carbon source; P is the PHA volumetric productivity.

**Table 6:** List of works concerning biocomposites formed by biopolymers and wine by-products as reinforcing fillers.

Matrix	Wine Waste	wt.% Range	Treatments of Fillers/Use of Coupling Agents	Ref
PHBV	Vine shoots	5–20	Tested both untreated and after polyphenol extraction	[34]
PHBV	Grape pomace	5–20	Tested both untreated and after polyphenol extraction	[34]
PHBV	Wine lees	10–30	Reactive extrusion with silane (for the 20 %wt. formulation)	[146]
PHBH	Wine lees	10–30	Reactive extrusion with silane (for the 20 %wt. formulation)	[146]
PHB	Wine lees	10–30	Untreated	[147]
PHBV	Grape pomace	5–20	Tested both untreated and after polyphenol extraction	[148]
PLA	Grape stalks	30–50	Untreated	[149]
PLA	Grape pomace	5–20	Untreated	[150]
PLA	Grape pomace	20–50	Reactive extrusion with maleic anhydride grafted PLA	[151]
PBS	Wine lees	10–30	Reactive extrusion with silane (for the 20 %wt. formulation)	[152]
PBS	Grape pomace	40–50	Reactive extrusion with maleic anhydride grafted PBS	[153]
PA11	Wine lees	10–30	Reactive extrusion with silane (for the 20 %wt. formulation)	[152]

**Table 7:** Physical and thermogravimetric analysis (TGA) data of the wine by-products present in the literature.

Wine Waste	Moisture [%]	Aspect Ratio	Particle Size [μm]	Density kg/m^3^	*T*_deg_ [°C]	Res [%]	*E*_f_ [GPa]	Ref
Vine shoots	-	Mixture of particles and long fibers	143	1.44	218	26	3.7–4.1	[34]
Grape pomace	-	Particles	114–121	1.42	181–215	32	2.7–2.8	[34,153]
Wine lees	48	Tiny particles	25	1.36–1.43	268	40	4.2–7.3	[146,152]
Grape stalks	22	Mixture of particles and long fibers	200–300	1.36	208–240	32–41	6.8–9.0	[149,155]
Grape seeds	24	Aggregates of particles	750	-	237	31	-	[155]
Grape peels	47	Particles	200–350	-	192	29.2	-	[155]

## Data Availability

Not applicable.
